# Prevalence and risk factors of cognitive frailty in people with HIV

**DOI:** 10.1097/QAD.0000000000004352

**Published:** 2025-11-24

**Authors:** Jovana Milic, Stefano Calza, Luca Lazzarini, Mattia Cocchi, Federico Motta, Stefano Renzetti, Laura Sighinolfi, Michela Belli, Vera Todisco, Maddalena Albertini, Altea Gallerani, Marianna Menozzi, Gianluca Cuomo, Giuseppe Mancini, Chiara Mussi, Cristina Mussini, Andrea Calcagno, Giovanni Guaraldi

**Affiliations:** a Department of Surgical, Medical, Dental and Morphological Sciences, University of Modena and Reggio Emilia, Modena; b Department of Molecular and Translational Medicine, University of Brescia, Brescia; c School of Medicine; d Department of Physical, Computer and Mathematical Sciences, University of Modena and Reggio Emilia, Modena; e Department of Medical-Surgical Specialties, Radiological Sciences and Public Health, University of Brescia, Brescia; f Department of Infectious Diseases, Azienda Ospedaliero-Universitaria, Policlinico of Modena, Modena; g Department of Surgical, Medical, Dental and Morphological Sciences, University of Modena and Reggio Emilia, Modena; h Unit of Infectious Diseases, Department of Medical Sciences, Amedeo di Savoia Hospital, University of Torino, Torino, Italy.

**Keywords:** cognitive frailty, frailty, HIV, neurocognitive function, neurocognitive impairment, people with HIV, polypharmacy

## Abstract

**Background:**

Cognitive frailty (CF, the simultaneous presence of frailty and cognitive impairment) is recognized as a significant predictor of several adverse health outcomes. The objective of this study was to describe prevalence and risk factors for CF in people with HIV (PWH) >50  years.

**Methods:**

This was a cross-sectional observational study including PWH attending Modena HIV Metabolic Clinic (MHMC). Neurocognitive function was measured with Cogstate battery that comprises six domains. Each individual CogState raw score was transformed into z-score after correction for age and sex. Neurocognitive impairment was defined by total global deficit score >0.5. Frailty was assessed by 37-Item frailty index. Scores <0.25 were considered fit or >0.26 as frail.

**Results:**

A total of 1258 PWH were included, 916 (73%) were males, median age was 58 years, median time since HIV diagnosis was 27 years. The sample was divided into four groups (CF) based on the presence of frailty (F) and cognitive impairment (ICT): F+/ICT+, F+/ICT−, F−/ICT+, F−/ICT−. Age per 5-year increase [odds ratio (OR) = 1.27, confidence interval (CI): 1.02–1.55, *P* = 0.022], nadir CD4^+^ cell count (OR = 0.81, CI: 0.66 – 0.99, *P* = 0.042) and polypharmacy (OR = 3.47, CI: 2.00–6.00, *P* < 0.001) were associated with CF after adjustment for time since HIV diagnosis, multimorbidity, depression and cumulative exposure to dolutegravir.

**Conclusion:**

CF prevalence in PWH >50 years was 6.8% and it is higher than what has been observed in the general population >65 years (1–4.4%). Nadir CD4^+^ cell count and polypharmacy was associated with CF, suggesting an HIV specific contribution related to the development of this condition.

## Background

Over the past two decades, the classification of HIV-associated neurocognitive disorders (HAND) has typically relied on the Frascati criteria [1]. However, alternative criteria have been proposed due to flaws in the HAND criteria. Advanced stages of HAND also involve mild to significant disability [1]. Given the changed spectrum of HIV disease, these criteria established in 2007, may result in an overestimation of disease burden and an ambiguity surrounding the underlying disease mechanisms [2]. Moreover, the minimum criteria for HAND assessment which are based solely on cognitive test performance, are not suitable for diverse populations with varying levels of education and socioeconomic backgrounds. This results in an overestimation of cognitive impairment that may heighten fear, stigma, and discrimination towards people with HIV [2].

The International HIV-Cognition Working Group, established in 2017 in response to methodological concerns with the HAND criteria, aimed to improve the diagnostic approach to cognitive impairment in people with HIV [3]. So far, an operational tool to characterize cognitive impairment in people with HIV that takes into consideration clinical history and acknowledges the multifactorial nature of low cognitive test performance is still not available [3].

It is clear that cognitive impairment should be considered in the context of the aging epidemic affecting both the general population and people with HIV in particular. To better understand the age-related increase in vulnerability, the term ”frailty” has been widely used over the past two decades to describe this mosaic of conditions caused by a reduction in homeostatic reserves, resulting in a higher risk of negative outcomes [4,5]. Frailty may also influence expression of neurocognitive impairment and it has been shown to predict future cognitive decline [6], suggesting that cognition and frailty may interact in advancing aging [7–9]. This is particularly clear with regards to brain pathology which may have a bidirectional association with frailty. It has been shown that brain aging and accumulation of common brain pathological findings (e.g. Alzheimer's disease) is associated with weakness and reduced gait speed [10–12], suggesting a shared pathological basis that may drive the simultaneous progression of both cognitive and physical decline, leading to the development of the cognitive frailty (CF) construct [13,14].

A decade ago, a consensus on CF definition was reached by an international consensus group from the International Academy of Nutrition and Aging (IANA) and the International Association of Gerontology and Geriatrics (IAGG) [13]. The proposed definition included the concurrent presence of physical frailty and cognitive impairment, without a contemporary diagnosis of dementia [13]. Moreover, it was recommended that all people with frailty should undergo an assessment for overall cognitive function. CF is recognized as a significant predictor of several adverse health outcomes, such as incident dementia, disability, falls and mortality [15–18]. However, it is still not clear which tools should be used for identification of cognitive frailty in the clinical setting [14,19].

So far, the CF construct has not been explored in people with HIV. In the coming decades, the number of people with HIV affected by CF is expected to increase as the population ages. The coexistence of physical frailty and cognitive impairment may have a synergistically negative impact on outcomes and quality of life, greater than either condition alone or the simple sum of their individual effects. We hypothesized that the describing this phenotype may provide a new operational framework to move on from HAND, promoting at a clinical level a patient centered approach and identifying a potential target for intervention that may help reverse adverse outcomes in older people with HIV.

The objective of this study was to describe prevalence and risk factors for CF in people with HIV >50 years.

## Methods

### Study design

This was an observational cross-sectional study that included antiretroviral therapy (ART)-experienced people with HIV attending Modena HIV Metabolic Clinic (MHMC) from January 2016 to April 2023. MHMC is a tertiary level referral center established in 2004 where people with HIV are screened for comorbidities, immuno-metabolic disorders, geriatric syndromes, neurocognitive impairment and frailty.

### Inclusion and exclusion criteria

People with HIV aged >50  years with both neurocognitive and frailty assessments were included. Neurocognitive and frailty evaluations are routinely preformed in all people with HIV on the same day as the visit in MHMC.

### Collected data and outcomes

Demographic, anthropometric, HIV-related variables including ART current and history of cumulative exposure and biochemical variables were collected on the same day of the visit at MHMC, including D-dimer and C-reactive protein. In particular, homeostasis model assessment of insulin resistance (HOMA-IR) was calculated using the following formula: HOMA-IR = [fasting glucose (mg/dl) × fasting insulin (mU/ml)]/405 [20].

Frailty was assessed with both frailty index and frailty phenotype. Frailty, assessed by 37-Item frailty index (FI) previously validated at MHMC, was built from health variables collected at the same study visit. Each variable included in the FI was coded with a value of 1 when a deficit was present, and 0 when it was absent. Missing values were removed from both the numerator and the denominator of the FI. The FI for each patient visit was calculated as the ratio between the number of deficits present and the total number of deficits assessed. We categorized people with HIV according to FI score as fit (<0.25) and frail (≥0.26). Each FI was computed when a minimum of 80% of valid data for the health variables was available [21]. Frailty phenotype was assessed using five established criteria: unintentional weight loss, self-reported exhaustion, weakness (grip strength), slow walking speed, and low physical activity [22]. PWH meeting 0 criteria were classified as fit, 1–2 as prefrail, and ≥3 as frail.

The assessment of cognitive performance was based on the Global Deficit Score (GDS) obtained through the Cogstate battery that evaluates six domains: simple speed processing, complex speed processing, attention/working memory, visual learning memory, verbal learning and verbal memory. Cogstate battery was selected on existing data demonstrating acceptable sensitivity and specificity for the screening purposes [23]. Supplementary Table 1, Supplemental Digital Content, summarizes the correspondence between CogState task and cognitive domains. Appendix 1, details how CogState battery is performed in clinical practice.

The population was divided into four groups based on the presence or lack of frailty (F) and impaired cognitive test (ICT).

Polypharmacy was defined as use of five or more drugs other than ART. Depression was defined according to Center for Epidemiologic Studies Depression Scale (CES-D-20), that ranges from 0 to 60 points. All scores higher than 16 were indicative of depression [24]. Comorbidities presented in Table 1 were defined according to the EACS guidelines [25]. Multimorbidity was defined as the presence of at least three comorbidities.

**Table 1:** Demographic, anthropometric and HIV characteristics, comorbidities and geriatric syndromes according to four frailty-cognitive performance phenotypes.

	Total *N* = 1258 (100%)	Cognitive frailty *N* = 85 (6.8%)	F+/ICT− *N* = 246 (19.5%)	F−/ICT+ *N* = 177 (14.1%)	F−/ICT− *N* = 750 (59.6%)	*P*
Demographic and anthropometric characteristics						
Age, years, median (IQR)	58.0 (7.4)	60.7 (8.6)	58.9 (7.5)	57.7 (8.6)	57.6 (7.2)	<0.001
Male sex, N (%)	916 (73%)	62 (73%)	189 (77%)	124 (70%)	541 (72%)	0.40
BMI, kg/m^2^, median (IQR)	24.5 (5.2)	27.5 (6.0)	26.8 (5.2)	23.7 (5.4)	23.6 (4.4)	<0.001
Obesity, *N* (%)	141 (11%)	24 (29%)	61 (25%)	14 (7.9%)	42 (5.6%)	<0.001
Sedentary lifestyle, *N* (%)	622 (52%)	36 (46%)	96 (41%)	85 (53%)	405 (57%)	<0.001
Intense smoking (>10 cigarettes/day), *N* (%)	191 (15%)	21 (25%)	49 (20%)	23 (13%)	98 (13%)	0.02
HIV characteristics						
HIV duration, months, median (IQR)	27 (11)	30 (12)	30 (9)	26 (11)	26 (11)	<0.001
Nadir CD4^+^ cell count, c/μl, median (IQR)	200 (210)	154 (210)	176 (185)	200 (245)	204 (203)	<0.001
Current CD4^+^ cell count, c/μl, median (IQR)	696 (379)	655 (427)	680 (382)	683 (373)	715 (364)	0.20
CD4/CD8 ratio, median (IQR)	0.95 (0.59)	0.87 (0.58)	0.90 (0.62)	1.03 (0.61)	0.96 (0.58)	0.02
Undetectable HIV RNA viral load, N (%)	223 (87%)	15 (75%)	54 (90%)	38 (90%)	116 (86%)	0.30
Current exposure to NNRTI, *N* (%)	410 (33%)	30 (35%)	76 (31%)	62 (35%)	242 (32%)	0.80
Current exposure to PI, *N* (%)	279 (22%)	22 (26%)	61 (25%)	29 (16%)	167 (22%)	0.20
Current exposure to INSTI, *N* (%)	847 (67%)	60 (71%)	174 (71%)	119 (67%)	494 (66%)	0.50
Cumulative exposure to NNRTI, years, median (IQR)	5.7 (9.2)	4.1 (6.6)	5.0 (9.2)	5.5 (9.1)	6.3 (8.6)	0.30
Cumulative exposure to PI, years, median (IQR)	7.8 (8.5)	4.7 (7.9)	7.1 (8.9)	6.8 (6.4)	9.1 (8.8)	0.30
Cumulative exposure to INSTI, years, median (IQR)	3.3 (5.4)	4.7 (6.4)	3.6 (5.3)	2.9 (5.5)	3.1 (5.2)	0.04
Cumulative exposure to RAL, years, median (IQR)	4.8 (6.9)	7.2 (8.1)	4.7 (6.6)	1.9 (5.3)	5.5 (6.5)	0.04
Cumulative exposure to DTG, years, median (IQR)	2.03 (4.04)	4.04 (4.21)	2.08 (3.29)	2.07 (4.25)	1.97 (2.85)	0.06
Multimorbidity and geriatric syndromes						
Multimorbidity, *N* (%)	310 (25%)	53 (62%)	92 (37%)	69 (39%)	96 (13%)	<0.001
AIDS-defining events, *N* (%)	160 (13%)	19 (22%)	35 (14%)	23 (13%)	83 (11%)	0.02
Cancer, *N* (%)	202 (16%)	21 (25%)	48 (20%)	28 (16%)	105 (14%)	0.03
Chronic kidney disease, *N* (%)	24 (1.9%)	5 (5.9%)	9 (3.7%)	4 (2.3%)	6 (0.8%)	<0.001
Cardiovascular disease, *N* (%)	93 (7.4%)	11 (13%)	23 (9.3%)	10 (5.6%)	49 (6.5%)	0.08
Chronic obstructive pulmonary disease, *N* (%)	57 (4.5%)	6 (7.1%)	13 (5.3%)	10 (5.6%)	28 (3.7%)	0.30
Diabetes, *N* (%)	248 (20%)	26 (31%)	81 (33%)	34 (19%)	107 (14%)	<0.001
Dyslipidemia, *N* (%)	1,025 (81%)	68 (80%)	214 (87%)	129 (73%)	614 (82%)	0.003
Hepatic cirrhosis, *N* (%)	146 (12%)	17 (20%)	44 (18%)	14 (7.9%)	71 (9.5%)	<0.001
Hypertension, *N* (%)	618 (49%)	51 (60%)	147 (60%)	67 (38%)	353 (47%)	<0.001
Lipodystrophy, *N* (%)	945 (75%)	62 (73%)	203 (83%)	124 (70%)	556 (74%)	0.02
Osteoporosis, *N* (%)	333 (26%)	25 (29%)	59 (24%)	48 (27%)	201 (27%)	0.70
Depression, *N* (%)	77 (8.4%)	5 (6.9%)	18 (10.1%)	9 (6.8%)	45 (8.4%)	0.70
Falls, *N* (%)	60 (15%)	4 (27%)	15 (23%)	5 (10%)	36 (13%)	0.06
Sarcopenia, *N* (%)	493 (39%)	44 (52%)	113 (46%)	78 (44%)	258 (34%)	<0.001
Polypharmacy, *N* (%)	250 (20%)	41 (48%)	100 (41%)	31 (18%)	78 (10%)	<0.001
Frailty phenotype, *N* (%)						
Fit	504 (42%)	17 (20%)	54 (23%)	72 (42%)	361 (50%)	<0.001
Prefrail	670 (55%)	60 (70%)	171 (73%)	93 (53%)	246 (47.8%)	
Frail	38 (3%)	8 (10%)	11 (4%)	8 (5%)	11 (2.2%)	

CF was used as on outcome and was defined as contemporary presence of frailty and impaired cognitive test (ICT). The rationale for including people with HIV aged 50 years and older (rather than only those over 65 years) derives from the consensus that individuals with HIV are considered older starting at age 50 [25]. The population was divided into four groups based on the presence or lack of frailty (F) and ICT. Therefore, the groups were: F+/ICT+, F+/ICT−, F−/ICT+, F−/ICT−.

### Statistical analysis

All CogState tasks’ results were obtained as standard scores and converted into *Z*-scores. *Z*-score (also called standard score) is a statistical way of determining the number of standard deviations that a raw score is either above or below the mean of a given population. So, in order to apply a *Z*-score, knowledge of both the population mean (*μ*) and standard deviation (*σ*) is required. The *Z*-scores range from −3 standard deviations, representing the extreme left of the normal distribution curve, to +3 standard deviations, which correspond to the extreme right of the curve.

Then, the formula shown in Supplementary Figure 1, Supplemental Digital Content, was used to calculate the impairment rate using the global deficit score (GDS) approach. Every domain was characterized by a specific formula that converted *Z*-score into a deficit score. All six deficit scores were combined and averaged to obtain the CogState GDS, which was considered impaired if ≥0.5. Supplementary Figure 2, Supplemental Digital Content, details how the GDS score was generated.

Data were summarized using median and interquartile range (IQR) for continuous variables and counts and percentages for categorical variables. Comparisons among groups were performed using Kruskal–Wallis ANOVA for continuous variables, and χ2 test for categorical variables. The association between risk factors of interest and CF at every visit was evaluated using a Generalized Linear Mixed Model (GLMM) assuming a binomial distribution (corresponding to a logistic model with multiple random effects) for the outcome (binary status C+/F+). All tests were two-sided, and assumed a 5% significance level. All the analyses were performed using R (version 4.2.2).

### Standard protocol approvals, registrations, and patient consent

Data complied fully with Italian law on personal data protection and the ethics committee of the Area Vasta Emilia Romagna Nord. The retrospective data were fully anonymized and the only sensitive data was year of birth. This was an observational study using de-identified data; therefore the IRB (Area Vasta Emilia Romagna Nord) did not require consent from the patient.

## Results

A total of 1258 people with HIV were included, 916 (73%) were males, median age was 58 (IQR: 7.4) years, median body mass index was 24.5 (IQR: 5.2) kg/m^2^, median time since HIV diagnosis was 27 (IQR: 11) years. HIV viral load was undetectable in 87% of people with HIV. The median CD4^+^ cell count was 696 (IQR: 379) c/μL and the mean CD4/CD8 ratio was 0.95 (IQR: 0.59) (Table 1).

**Fig. 1:**
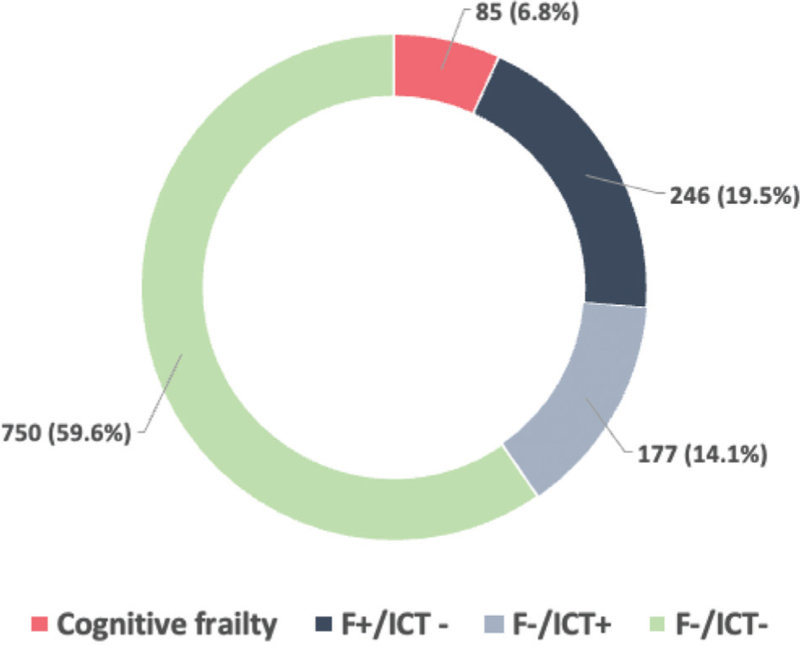
Prevalence of four frailty/impaired cognitive test.

As mentioned before, the population was divided into four groups based on the presence or lack of frailty (F) and impaired cognitive test (ICT). Therefore, the groups were: F+/ICT+, F+/ICT−, F−/ICT+, F−/ICT− (Fig. 1). CF was present in 85 (6.8%) and incidence was 2.9 x 100 persons/years.

People with CF were older (median age 60.7 years) than people in F+ICT− (58.9), F−ICT+ (57.7), and F−ICT− (57.6) group (*P* < 0.001). They also had higher BMI (CF: 27.5 kg/m^2^ vs. F+/ICT−: 26.8, F−/ICT+: 23.7, F−/ICT−: 23.6; *P* < 0.001), higher prevalence of obesity (CF: 29% vs. F+/ICT−: 25%, F−/ICT+: 7.9%, F−/ICT−: 5.6%; *P* < 0.001) and smoking >10 cigarettes a day (CF: 25% vs. F+/ICT−: 20%, F−/ICT+: 13%, F−/ICT−: 13%; *P* = 0.016). Surprisingly, a sedentary lifestyle was less frequent in frailty groups (CF: 46%, F+/ICT−: 41%, F−/ICT+: 53%, F−/ICT−: 57%; *P* < 0.001) (Table 1).

Regarding HIV-related variables and ART, people with CF had longer time since HIV diagnosis (CF: 30 years vs. F+/ICT−: 30, F−/ICT+: 26, F−/ICT−: 26; *P* < 0.001), lower CD4^+^ nadir (CF: 154 c/μl vs. F+/ICT−: 176, F−/ICT+: 200, F−/ICT−: 204; *P* < 0.001), lower % current CD4^+^ cell count (CF: 32% vs. F+/ICT−: 33, F−/ICT+: 36, F−/ICT−: 36; *P* = 0.007) and lower CD4/CD8 ratio (CF: 0.87 vs. F+/ICT−: 0.90, F−/ICT+: 1.03, F−/ICT−: 0.96; *P* = 0.016), when compared to other groups. Cumulative, but not current, exposure to raltegravir, was higher in people with CF (CF: 87 months vs. F+/ICT−: 57, F−/ICT+: 23, F−/ICT−: 66; *P* = 0.04). Cumulative exposure to dolutegravir was also higher in people with CF (CF: 48 months vs. F+/ICT-: 25, F−/ICT+: 25, F−/ICT−: 24; *P* = 0.06). Other HIV-related variables and ART were not associated with CF or with any of the other three groups (Table 1).

As expected, multimorbidity was significantly higher in people with CF (CF: 62% vs. F+/ICT−: 37%, F−/ICT+ : 39%, F−/ICT−: 13%; *P* < 0.001). Single comorbidities, such as cancer, chronic kidney disease, diabetes, dyslipidemia, hepatic cirrhosis, hypertension, lipodystrophy, were associated with CF (Table 1).

With regards to geriatric syndromes and frailty phenotype, prevalence of polypharmacy was the highest in people with CF (CF: 48% vs. F+/ICT−: 41%, F−/ICT+: 18%, F−/ICT−: 10%; *P* < 0.001). Frailty and prefrailty, assessed with frailty phenotype, were the highest among people with CF. Sarcopenia, assessed with hand grip, was higher in people with impaired cognitive performance (CF: 52% vs. F+/ICT−: 46%, F−/ICT+: 44%, F−/ICT−: 34%; *P* < 0.001). Other geriatric syndromes, such as falls, were higher in people with HIV with CF (CF: 27% vs. F+/ICT−: 23%, F−/ICT+: 10%, F−/ICT−: 13%; *P* = 0.056), although statistically not significant (Table 1).

**Fig. 2:**
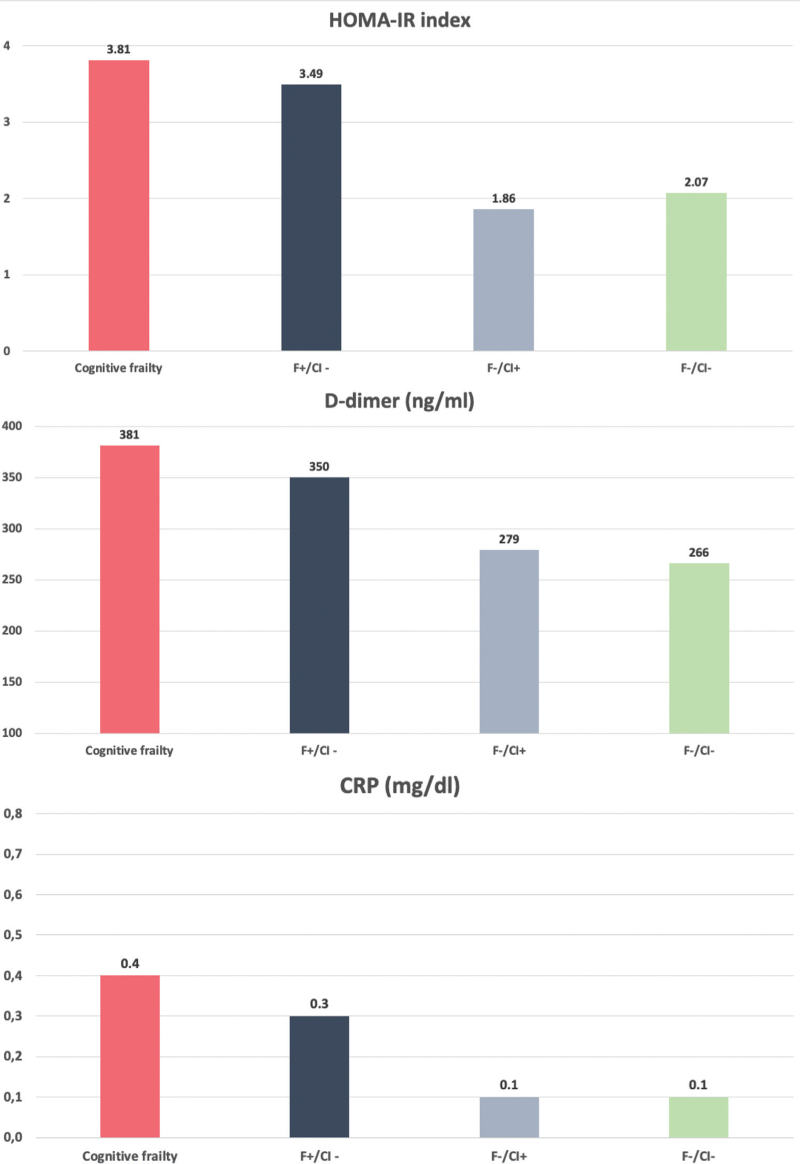
Meta-inflammatory biomarkers according to four frailty/impaired cognitive test.

Meta-inflammatory biomarkers were higher in frailty groups, in particular in people with cognitive frailty. In detail, CRP (CF: 0.40 mg/dl vs. F+/ICT−: 0.30, F−/ICT+: 0.10, F−/ICT−: 0.10; *P* < 0.001), D-dimer (CF: 381 ng/ml vs. F+/ICT−: 350, F−/ICT+: 279, F−/ICT−: 266; *P* < 0.001) and HOMA-IR index (CF: 3.81 vs. F+/ICT−: 3.49, F−/ICT+: 1.86, F−/ICT−: 2.07; *P* < 0.001) were higher in people with CF (Fig. 2).

**Fig. 3:**
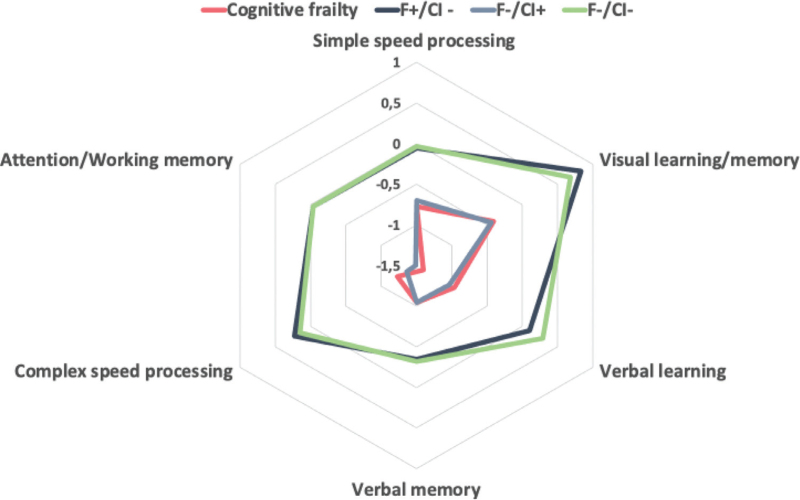
Six cognitive domains according to four frailty/impaired cognitive test.

Figure 3 shows that people with CF had lower neurocognitive performance at all six domains when compared to other three groups (all *P* < 0.001).

In GLMNM model, age (OR = 1.27, CI: 1.02–1.55, *P* = 0.022), nadir CD4^+^ cell count (OR = 0.81, CI: 0.66–0.99, *P* = 0.042) and polypharmacy (OR = 3.47, CI: 2.00–6.00, *P* < 0.001) were associated with CF after adjustment for time since HIV diagnosis, multimorbidity, depression and cumulative exposure to DTG (Table 2A).

**Table 2:** Logistic regression models for cognitive frailty including cumulative exposure to dolutegravir (DTG) (panel A) and cumulative exposure to raltegravir (RAL) (panel B).

Panel A			Panel B		
Variable	OR (95% CI)	*P*	Variable	OR (95% CI)	*P*
Cumulative exposure to DTG	1.06 (0.94–1.18)	0.311	Cumulative exposure to RAL	1.03 (0.94–1.12)	0.487
Age^a^	1.27 (1.03–1.55)	0.022	Age^a^	1.27 (1.03–1.55)	0.023
Nadir CD4^+^ cell count	0.81 (0.66–0.99)	0.042	Nadir CD4^+^ cell count	0.81 (0.66–0.99)	0.044
Time since HIV diagnosis^a^	1.02 (0.87–1.22)	0.799	Time since HIV diagnosis^a^	1.03 (0.87–1.22)	0.769
Multimorbidity	0.55 (0.26–1.25)	0.132	Multimorbidity	0.56 (0.27–1.28)	0.145
Polypharmacy	3.47 (2.00–6.00)	<0.001	Polypharmacy	3.44 (1.98–5.95)	<0.001
Depression	1.00 (0.97–1.03)	0.786	Depression	1.00 (0.97–1.03)	0.894

a 5 years increase.

In GLMM model, age (OR = 1.27, CI: 1.03–1.55, *P* = 0.023), nadir CD4^+^ cell count (OR = 0.81, CI: 0.66 – 0.99, *P* = 0.044) and polypharmacy (OR = 3.44, CI: 1.98–5.95, *P* < 0.001) were associated with CF after adjustment for time since HIV diagnosis, multimorbidity, depression and cumulative exposure to RAL (Table 2B).

## Discussion

The objective of this study was to provide a description of cognitive frailty in people with HIV, defining its prevalence in patients aged >50 years, risk factors and association with polypharmacy. This study shows the prevalence of cognitive frailty of 6.8%. The data in the general population regarding its prevalence is discordant [15]. In a nonconsistent body of evidence, prevalence of CF varies from 1% to 22% with hospital settings tend to have higher prevalence, ranging from 10.7% to 22%. In contrast, population-based studies have shown lower prevalence rates, generally being between 1.0% and 4.4%. All these studies consider a geriatric population, aged over 65 years.

The high prevalence of CF in people with HIV > 50 years can be justified by accelerated aging profile with the burden of chronic comorbidities, multimorbidity and frailty, being similar to what is observed in people without HIV 10 years older [26,27]. CF may be multifactorial in nature and includes both HIV specific and traditional risk factors. An accelerated aging condition is sustained by chronic inflammation, originating from the interplay of inflammaging, immunosenescence and immunometabolism. In the general population, as in people with HIV, it has been demonstrated that increased levels of D-dimer and CRP are associated with frailty [28]. In our study, both D-dimer and CRP were also associated with CF, suggesting common pathways involved in the development of frailty and cognitive impairment in people with HIV.

D-dimer is an endothelial biomarker of inflammation and coagulation, and it has been shown to be associated with serious non-AIDS events (cardiovascular disease, cancer, serious renal and hepatic disease) and mortality in virally suppressed people with HIV with moderate to high CD4^+^ cell counts [29]. The relationship between immune activation and cognition in people with HIV is complex. Chronic immune activation, even in virologically suppressed people with HIV, has been associated with cognitive impairment. Monocyte activation markers such as soluble CD163 (sCD163) and soluble CD14 (sCD14) have been linked to worse cognitive performance across various domains including verbal learning, memory, and executive function [30]. It should be acknowledged that D-dimer is a nonspecific marker that can be influenced by various conditions and may not reliably reflect chronic inflammation. In contrast, fibrinogen has been identified in multiple studies as a marker of chronic low-grade inflammation in the context of frailty [31]. However, since fibrinogen is not routinely measured at MHMC, its association with CF could not be evaluated in this study.

While CF is not intended to replace the HAND classification [1], it may offer a broader framework to capture the complex interplay of physical frailty and cognitive impairment in aging people with HIV. The HAND construct primarily focuses on cognitive dysfunction attributed to HIV and includes disability in its more advanced stages, whereas newer classifications such as brain injury in people with HIV includes HIV-associated brain injury (HABI), but also acknowledges a wider range of neuropathological contributors [2]. In this evolving landscape, CF may serve as a complementary construct that supports a multidimensional approach to care.

To avoid misinterpretation or overstatement, this study did not aim to diagnose cognitive impairment but instead used the term “impaired cognitive test,” as assessed by the Cogstate battery. Although Cogstate is not commonly used in the general population, it was chosen based on existing evidence supporting its use in people with HIV [23]. Additionally, the EACS guidelines do not specify which neurocognitive battery should be employed [25]. On the other hand, we considered frailty index to be more suitable for estimating cognitive frailty, as it captures a broader range of age-related deficits across multiple domains compared to the frailty phenotype, which is also routinely collected in our cohort. Nevertheless, frailty, measured by either frailty index or frailty phenotype, is associated with worse global neurocognitive functioning [32–34]. Moreover, frailty in people with HIV is associated with lower CD4^+^ T-cell counts, detectable viral load [35] and immune activation markers such as the CD4/CD8 ratio and nadir CD4^+^ cell count, suggesting that immune dysregulation plays a role in the development of frailty [36]. Additionally, frailty and HIV disease severity have been shown to synergistically increase the risk for neurocognitive impairment [37]. In this scenario, frailty may act as a modifier of disease expression and progression, and its assessment should be integrated into the management of people with cognitive impairment to inform treatment decisions and improve patient outcomes.

Our results also imply that subclinical metabolic conditions, such as insulin resistance, is associated with CF. Insulin resistance is related to frailty, independently of sarcopenia and cognitive impairment [38,39]. Insulin resistance may expose cells, including neurons, to high insulin levels for an extended period, which can negatively impact their function and survival [40]. In people with HIV, insulin resistance has been linked to lower cognitive performance, with studies showing that higher levels of HOMA are associated with poorer performance on neuropsychological tests [41]. This relationship persists even when controlling for factors such as age, CD4^+^ cell count, and antiretroviral therapy [42].

In our study, both multimorbidity and polypharmacy were related to CF. The majority of medications were prescribed for the management of comorbidities, but we cannot exclude the potential contribution of medications prescribed for other indications, such as proton pump inhibitors, corticosteroids, or treatments for sexual dysfunction. The presence of multiple comorbidities and the associated polypharmacy represent a common pathogenetic mechanism for both cognitive impairment and frailty. These mechanisms may be complex and bidirectional. Frailty is linked to certain chronic diseases and multimorbidity, which can consequently lead to polypharmacy. On the other hand, there are plausible mechanisms by which drugs may affect the development of frailty. Several components of frailty have been directly correlated with medication burden, with potential impact on weight loss, balance disorders, poor nutritional status, or functional deterioration [43]. In clinical practice, it is important to monitor for signs of frailty and cognitive impairment in people with HIV, as these conditions may compound and lead to greater functional decline. Interventions to prevent or reduce frailty may be beneficial in maintaining cognitive health in this population [44,45]. Interventions may also include physical and cognitive training programs, nutritional counseling, and psychosocial therapies, which have shown promise in reducing the risk of adverse health outcomes [46]. The use of freely available, easy-to-use tools for screening cognitive impairment or frailty should help integrate CF screening into clinical practice.

The relationship between depression and both frailty and neurocognitive impairment is well known in people with HIV. Depression is associated with overall cognitive impairment, affecting multiple domains, such as processing speed, learning and memory and executive function [47]. On the other side, depression may lead to decreased physical activity, exhaustion and poor nutrition, contributing to the development of frailty [48]. In the general population, CF is related to two-fold higher risk of depression, with prevalence of depression among persons with CF around 46% [49]. However, our study did not find a link between depression and CF, possibly because the tools used to assess both cognitive performance and depression are primarily designed as screening tools rather than for definitive diagnosis.

Our study did not identify the relationship between ART and CF, although there was an initial signal that INSTI and in particular RAL may be associated with CF. Direct effects of specific ART regimens on CF are not clarified. Specific ART classes have been associated with different cognitive outcomes. Women living with HIV exposed to nonnucleoside reverse transcriptase inhibitors demonstrated improvements in verbal learning compared to other treated women, while those exposed to protease inhibitors had worse verbal learning and verbal memory performance at baseline compared to women without HIV, although they showed improvements over time [50]. Some studies have reported poorer cognitive outcomes associated with INSTI use, particularly with certain agents within this class. Specifically, the use of dolutegravir and elvitegravir, but not raltegravir, has been associated with poorer learning outcomes in women with HIV [51]. Another study found differences in learning/memory performance and smaller brain volumes in people with HIV on INSTI-based regimens compared with non-INSTI users [52]. Additionally, longer lifetime exposure to INSTIs, especially dolutegravir, was identified as an important predictor of neurocognitive impairment [53]. Conversely, other research has shown that neuropsychological performance improved regardless of INSTI use, with an attenuation of improvement in verbal memory in the postswitch versus preswitch period, suggesting that INSTI did not have a consistent detrimental effect on neuropsychological outcomes [54].

Some limitations are intrinsic to the cross-sectional nature of the study that cannot assess causal relationships between the variables. Secondly, our population might be still too young to capture associations between cognitive frailty and traditional geriatric outcomes, such as falls and disability. Thirdly, we were not able to evaluate anticholinergic burden, drug-drug interactions and potentially inappropriate prescription that might diversely impact cognitive frailty and other three explored phenotypes. Fourthly, our results might not be generalizable to other countries and regions with different HIV models of care and management of people with HIV. Fifthly, our polypharmacy definition did not include ART, therefore potential biases related to drug-drug interactions, inappropriate prescription and anticholinergic burden cannot be accounted for. Finally, the models applied in the study rely on the usual assumptions and unmeasured confounding cannot be ruled out.

However, some strengths should be acknowledged. MHMC is a large and well characterized cohort that collects data on lifestyles and comprehensive geriatric assessment. To our knowledge, this is the first study in the HIV setting that described CF in the context of polypharmacy.

In conclusion, CF prevalence in people with HIV > 50 years was higher to what was observed in the general population >65  years. Nadir CD4^+^ cell count was associated with CF, suggesting an HIV specific contribution along with chronic inflammation related to the development of this condition.

## Acknowledgements

Authors’ contributions: J.M., A.C. and G.G. conceptualized and designed the manuscript. J.M. and G.G. wrote and revised the manuscript. S.C., F.M. and S.R. did the statistical analysis. J.M., S.C., ChiM, CriM, A.C. and G.G. did the supervision of the final version of the manuscript. All the authors contributed to discussion and revised the manuscript.

Funding: This paper did not receive any funding.

### Conflicts of interest

J.M. received speaker honoraria from Gilead and ViiV. G.G. and C.M. received research grant and speaker honoraria from Gilead, ViiV, MERCK, Jansen and Pfizer. G.G. and C.M. attended advisory boards of Gilead, ViiV and MERCK.

## Supplementary Material

Supplemental Digital Content

## Supplementary Material

Supplemental Digital Content
