# Supplementary material for: Prevalence and risk factors of cognitive frailty in people with HIV
Source: AIDS. 2025 Nov 24;40(2):133–42. doi: 10.1097/QAD.0000000000004352 (PMC12746784; doi:10.1097/QAD.0000000000004352)

***Supplementary table 1*** *summarizes the correspondence between CogState task and cognitive domains.*

| **Cognitive domains** | **CogState Tasks** |
| --- | --- |
| Simple speed processing | Detection |
| Complex speed processing | One-back speed |
| Attention/ Working Memory | One-back accuracy |
| Visual learning/memory | One Card Learning accuracy |
| Verbal learning | International shopping List - total Learning |
| Verbal memory | International Shopping - List Delayed Recall |

***Supplementary figure 1.*** *Conversion of Z-scores for each domain into a deficit score. In this figure, z-scores for detection are used. The same model is applicable to all other five domains.*


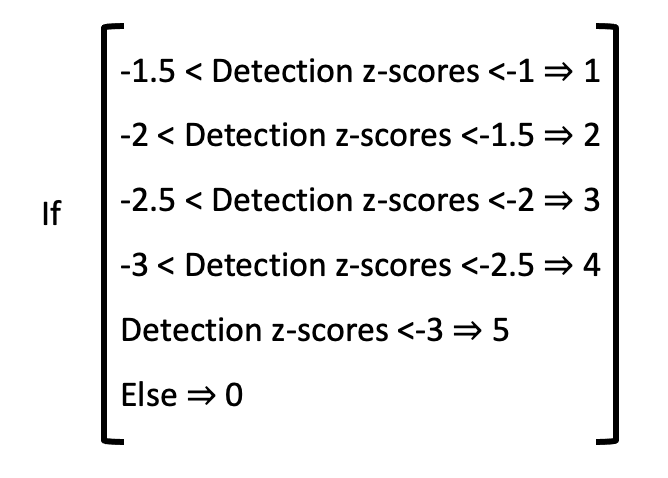


***Supplementary figure 2.*** *Global deficit score (GDS) calculations from CogState.*


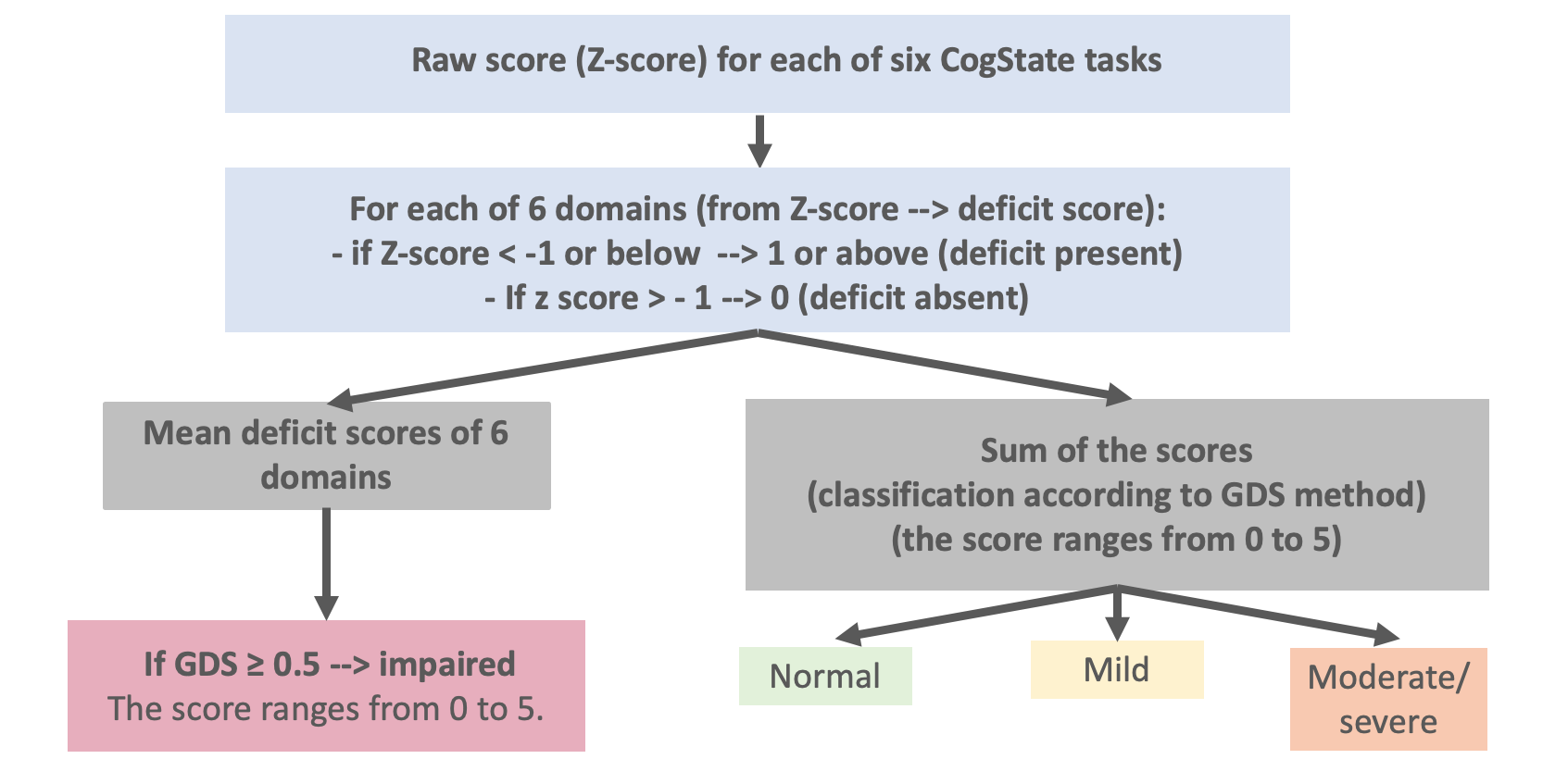

Supplement: Supplemental Digital Content [file aids-40-133-s001.docx]
