# Supplementary material for: Prevalence and risk factors of cognitive frailty in people with HIV
Source: AIDS. 2025 Nov 24;40(2):133–42. doi: 10.1097/QAD.0000000000004352 (PMC12746784; doi:10.1097/QAD.0000000000004352)
Supplement: Supplemental Digital Content [file aids-40-133-s002.docx]

**Appendix 1**

The application of the CogState battery was carried out at the moment of the visit using a personal computer. The battery included four different tasks to assess the six neurocognitive domains aforementioned. The psychologist had the role to explain the task to the patient and was available to assist the participants in comprehending the assignments throughout the practice session. However, during the testing process, the psychologist offered only limited guidance and oversight. The four tasks were administered in the subsequent order: (i) International Shopping List Test (ISLT) exploits a 12-word list learning paradigm to assess verbal learning. The participant is read three times a shopping list and must remember and recall as many items from the list as possible. The International Shopping List Test is also performed at the end of the examination, as a “Delayed Recall” version, which measures verbal memory, when the participant is asked to repeat the list. For the International Shopping List Test, the tablet is held by the test administrator and the screen is not visible to the test participant; (ii) The Detection test measures processing speed using a simple reaction time paradigm. The on-screen instructions ask: “Has the card turned over?”. A playing card is presented face down in the center of the screen. The card flips over so it is face up. As soon as the card flips over the participant must press “Yes”. The participant is encouraged to work as quickly as they can and be as accurate as possible; (iii) One Card Learning Test (OCL) measured visual memory through a pattern separation paradigm. A single card was displayed facing upwards at the center of the screen, and the individual was prompted to determine whether or not they had previously encountered the card in the current test. The measured outcome was represented by the speed of performance; (iv) One Back speed (ONB) assessed working memory. A playing card was displayed facing upwards at the center of the screen. The task for the participant was to determine whether this card matches the previous one or not. The performance speed was calculated.
